# Biological, Biochemical and Elemental Traits of *Clavelina oblonga*, an Invasive Tunicate in the Adriatic Sea

**DOI:** 10.3390/ani15101371

**Published:** 2025-05-09

**Authors:** Natalija Topić Popović, Bojan Hamer, Ivančica Strunjak-Perović, Tibor Janči, Željka Fiket, Matilda Mali, Luca Privileggio, Kristina Grozić, Dijana Pavičić-Hamer, Lucija Vranjković, Tamara Vujović, Marija Miloš, Maria Michela Dell’Anna, Darya Nefedova, Rozelindra Čož-Rakovac

**Affiliations:** 1 Laboratory for Aquaculture Biotechnology, Ruđer Bošković Institute, Bijenička 54, 10000 Zagreb, Croatia; ntopic@irb.hr (N.T.P.); strunjak@irb.hr (I.S.-P.); p_lucija@yahoo.com (L.V.); tamara.vujovic@irb.hr (T.V.); maja.bakovic@hotmail.com (M.M.); rrakovac@irb.hr (R.Č.-R.); 2 Laboratory for Marine Nanotechnology and Biotechnology, Center for Marine Research, Ruđer Bošković Institute, Giordano Paliaga 5, 52210 Rovinj, Croatia; luca.privileggio@irb.hr (L.P.); kristina.grozic@irb.hr (K.G.); pavicic@cim.irb.hr (D.P.-H.); 3 Faculty of Food Technology and Biotechnology, University of Zagreb, Pierottijeva 6, 10000 Zagreb, Croatia; tjanci@pbf.hr; 4 Laboratory for Inorganic Environmental Geochemistry and Chemodynamics of Nanoparticles, Ruđer Bošković Institute, Bijenička 54, 10000 Zagreb, Croatia; zeljka.fiket@irb.hr; 5 Department of Civil, Environmental, Land, Building Engineering and Chemistry, Polytechnic University of Bari, Via E. Orabona 4, 70126 Bari, Italy; matilda.mali@poliba.it (M.M.); mariamichela.dellanna@poliba.it (M.M.D.); darya.nefedova@poliba.it (D.N.)

**Keywords:** ascidian tunicate, mussel farming, biofouling, biomass, proximate composition, fatty acids, elemental analysis, environmental conditions, Lim Bay, Mediterranean area

## Abstract

*Clavelina oblonga* is an invasive tropical tunicate recently introduced into the Adriatic Sea as a consequence of globalization and climate change. Mussel aquaculture sites provide an ideal environment for this colonial ascidian, where it has recently become the dominant fouling species. This study represents the first investigation of its biological and physical characteristics, as well as its proximal, fatty acid, macroelement, trace element, and toxic metal composition. The entire-tissue chemical composition of *C. oblonga* resulted in 95.44% moisture. Its composite structure revealed several strong peaks, attributed to O-H, C-H, C-N, and C=O stretching, along with cellulose components overlapping with proteins and carbohydrates. The major fatty acids were palmitic, stearic, and docosahexaenoic acid, followed by docosanoic, elaidic, linoleic, and myristic acid. The saturated fatty acids, polyunsaturated fatty acids, and monounsaturated fatty acids comprised 51.37, 26.96, and 15.41% of the total fatty acids, respectively. Among the analysed trace and macroelements, aluminium and sodium were predominant. *C. oblonga* exhibited different concentrations of toxic metals, such as arsenic and lead, compared to fouled mussels in the Istria region. It appears that the tunicate has adapted to the environmental conditions of the Adriatic, reaching its maximum spread and biomass in mid-autumn. There is a strong possibility that *C. oblonga* could colonize and establish itself permanently in the Adriatic. This would have a strong negative impact on shellfish farming, the structure of the ecosystem, plankton biomass, and the distribution of other marine species. However, it also represents a biomass resource with high potential of utilization in different industries.

## Simple Summary

*Clavelina oblonga* is a non-native marine organism that was recently introduced into the Adriatic Sea. The spread of this species, which belongs to a group of animals called ascidian tunicates, in new areas is likely related to human activities and climate change. *C. oblonga* proliferates and grows rapidly in mariculture areas where mussels are cultivated on culture ropes. In such areas, it has become one of the most common biofouling species, attaching itself to mussels and other man-made surfaces. This study examined its biological and biochemical properties by analyzing its moisture content, fatty acids, minerals, and some less common elements. The results of this study showed that *C. oblonga* contains different levels of certain toxic metals, such as arsenic and lead, when compared to mussels that it overgrows. On-site observations indicate that *C. oblonga* has adapted to the colder temperatures of the northern Adriatic Sea and may continue to spread, potentially disrupting shellfish farming, reducing plankton populations, and altering marine ecosystems. The large quantities of biomass produced by this species could be used in various industries, making it essential to understand the overall characteristics of *C. oblonga* for that purpose.

## 1. Introduction

Tunicates are remarkably diverse species, with close to 3000 species worldwide [1], subdivided into the classes Thaliacea (free-swimming colonial organisms), Appendicularia (free-swimming solitary organisms), and Ascidiacea (sea squirts; their sessile adults attach to solid substrates) [2]. Ascidians are predominantly hermaphroditic, sometimes capable of self- and cross-fertilization, with the ability to form new individuals by budding [3]. As omnivorous filter feeders, vigorous competitors for habitat and food, and rapid colonizers of available surfaces, tunicates are a significant ecological threat as an invasive species [4]. Tunicates contract their circular muscles to pump seawater through oral siphons and filter it for feeding on plankton and particles ranging from 1 µm to 1 mm in size. They can filter water at rates exceeding 1000 times their body volume, with a filtration rate of 15.3 mL/s [1], thus having impacts that extend beyond their physical dominance on the substrate. Tunicates can affect plankton communities in the seasons when their abundance and filtration rates are at their highest [5]. The introduction of non-native ascidians is associated with negative ecological impacts through outcompeting native species of benthic communities and detrimental effects on the aquaculture industry [4]. As ascidians reproduce and grow rapidly, have a short maturity period, a simplistic feeding mode, and often possess anti-predator defences and spread readily, they are endowed with characteristics common to flourishing invasive organisms [1,6].

Ascidians are frequently found in the Mediterranean Sea, where *Clavelina oblonga* is widespread and already established [7,8]. It is native to the tropical southern Atlantic coast of North America and the Caribbean Sea, but has been introduced to Brazil, the Azores, Cape Verde, Senegal, and the Mediterranean Sea [8]. *C. oblonga* is a species with brownish, transparent tunics forming large spherical colonies [9]. The colonies are united by stolons, forming clusters. Their thorax and abdomen regions are up to 20–30 mm long, with white flecks on the tunic, while the branchial sac has 15–18 rows of stigmata [10,11]. The growth of *C. oblonga* is related to the increases in seawater temperature and food availability [7].

Anatomically, all ascidians consist of two main and distinct body parts: the tunic and the remaining internal organs. The tunic, which performs various biological functions, comprises the external supportive and protective skeleton as well as the integumentary tissues. It has long been known to contain approximately 60% cellulose and 27% nitrogen-containing components by dry weight [8,12]. The inner body tissues include all body parts covered by the tunic, including the pharyngeal basket, endostyle, testis, ovary, heart, stomach, intestine, and anus, among others, which serve different functions such as feeding, digestion, excretion, and other essential life processes. The inner body primarily consists of crude proteins, with a much smaller amount of carbohydrates and a higher amount of lipids. The mass content of the main body components (carbohydrates, proteins, lipids, and ash) varies among different ascidian species [12].

Although previously recorded in several locations of the northern Adriatic Sea, a highly intense *C. oblonga* invasion occurred in the summer of 2020 in the shellfish along the western coast of the Istrian peninsula (Croatia). *C. oblonga* has apparently adapted to colder conditions, surviving and thriving over six winters in the northern Adriatic [13], where winter sea temperatures exceed 10 °C [14]. Previous studies reported that the lowest sea surface temperature at which *C. oblonga* remains active is 11.2 °C [7]. Its further spread in the northern Adriatic is expected [11], particularly given its successful overwintering, as zooids (young, active specimens) were detected in February in both deeper and upper water layers, attached to old colonial buds [13].

Its invasion of the Adriatic Sea and throughout the rest of the Mediterranean is facilitated by anthropogenic activities, globalized maritime trade, ballast water, and sediments in ballast tanks and on boat hulls [9,15]. As they are short-lived in the water column, larvae cannot disperse over great distances without human-mediated transport. Their survival during transport and invasion is enabled by their capacity to colonize both degraded and nutrient-rich environments [7,16]. Artificial substrates and solid matter are important habitats for *C. oblonga* before and after its establishment, a process further supported by rising sea temperatures [11,17]. *C. oblonga* poses significant threats to mariculture facilities through biofouling of bivalve cultures, adding weight to load-bearing infrastructure, and competing with bivalves for food. It frequently overgrows bivalves, restricts water exchange and nutrient inflow, preys on their larvae and excludes juveniles, interferes with the secretion of foot filaments, hinders shell opening, and affects feeding and respiration, thus increasing shellfish mortality and decreasing productivity [1,8,18].

Intensive biofouling by *C. oblonga* increases mussel production costs due to the need for regular mechanical or chemical cleaning of culture ropes and man-made infrastructure, while minimizing the reintroduction of tunicate fragments into the environment [13,19]. In non-native areas, the removal of *C. oblonga* could be considered part of a mandatory, even if economically demanding, strategy for managing invasive alien species [19]. Although the ecosystem disservices caused by the *C. oblonga* invasion are relevant and well-documented [8,13,19], there is limited understanding of the relationship between invasive tunicate species and the ecosystem services they could provide [12,20]. The large quantities of biomass produced, even if highly undesirable, could potentially serve as a valuable and sustainable resource for various sectors [20,21].

In the northern Adriatic Sea, a semi-enclosed sea in the northernmost part of the Mediterranean, Lim Bay is an area of intensive production of the mussel species *Mytilus galloprovincialis*. It is one of the most important mussel and oyster mariculture areas along the entire Croatian Adriatic coast [22], and is severely affected by the *C. oblonga* invasion. Therefore, to gain insight into *C. oblonga* traits in Lim Bay, we aimed to investigate its presence, temporal dynamics, and biological properties, along with its biochemical and physical characteristics (proximate composition, fatty acid composition, composite structure, and thermogravimetry), as well as its elemental constituents. The primary objective of this study was to develop a fundamental understanding of this largely understudied organism, which increases farming costs and causes losses in mussel production. Additionally, the study aimed to compare the obtained data with literature reports on other ascidians.

## 2. Materials and Methods

### 2.1. Study Site

**Figure 1:**
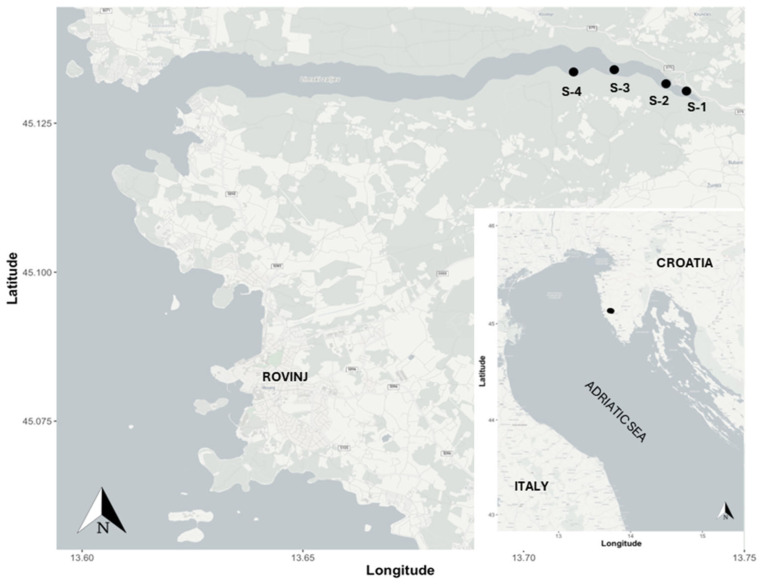
Map of the investigated mussel farming area in Lim Bay and *Clavelina oblonga* overgrowth sampling sites (S-3 and S-4), Adriatic Sea, Croatia.

The study was conducted in 2023 on the invasive tunicate *Clavelina oblonga* Herdman, 1880 (Ascidiacea, Aplousobranchia, Clavelinidae), which was collected from a mussel farm on the western coast of the Istrian peninsula. Specifically, the sampling sites were located in Lim Bay, situated in the northern Adriatic Sea, Croatia, at coordinates S-3 (45.13447° N, 13.71766° E) and S-4 (45.13364° N, 13.47113° E) (Figure 1).

Lim Bay, situated 5 km north of Rovinj, Croatia, is an 11 km long semi-enclosed marine inlet, resembling a fjord, on the west coast of Istria, along the Adriatic Sea. Its unique and isolated geographical position has fostered the development of endemic marine flora and fauna, as well as a longstanding tradition of shellfish farming. Recognized as a marine protected area since 1967, Lim Bay is designated as a special underwater reserve [22]. The bay features distinct variations and gradients in ecological parameters across different sites from east to west, primarily influenced by freshwater inflow, which introduces variations in nutrients, temperature, oxygen content, salinity, and water current velocity. This dynamic interplay contributes to the habitat diversity within Lim Bay [23,24,25]. Within this environment, mussels are cultivated on ropes suspended from rafts buoyed by plastic floats. Each raft accommodates approximately 30 rope nets, each containing mussels and extending 2–6 m in length. The mariculture farm under investigation produces approximately 50 t of *M. galloprovincialis* annually [26].

During the sampling of *C. oblonga* biomass for this study (Supplementary Figure S1a–f), we conducted a comprehensive water column profiling. This involved measuring temperature, conductivity/salinity, dissolved oxygen, and fluorescence/chlorophyll-a. We employed the AquaTroll 500 CTD multiparameter probe (InSitu, Fort Collins, CO, USA) to describe the environmental conditions during the sampling period of *C. oblonga*. In addition, to obtain continuous data, temperature readings taken every 15 min were collected at the depth of 5 m. The data loggers used for this purpose were HOBO MX2203 and U24-002-C (Onset, Bourne, MA, USA), covering the period from 2021 to 2023 (Supplementary Figure S2a–e).

### 2.2. Samples

Specimens of *C. oblonga* were first recorded and identified in the NE Adriatic Sea in 2015 during a national survey. Subsequent monitoring activities were conducted across the entire peninsula in the following years, with particular focus on areas with established presence [13]. A total of 150 colonies (2 × 50 L, >100 kg) of *C. oblonga* were collected from the mussel farming ropes (sites S-3 and S-4) in the fall (October 2023). Colonial zooids were manually detached as the ropes were pulled out of the sea, placed on a flat surface, and quickly processed to separate the *C. oblonga* fouling from the mussels and ropes. Since the mussels were encased in a net to protect against fish predation, colonies were collected only on ropes where *C. oblonga* formed spheres up to 5–30 cm in diameter (Supplementary Figure S1a–f; Supplementary Video S1).

Upon collection, the colonial zooids were washed with seawater, counted, wet-weighed, examined for external mechanical damage, separated, sorted, placed in sealed PE bags, transported to the laboratory on ice, and frozen at –86 °C (model ULF50086, Infrico medcare, Cordoba, Spain) until further analyses. Prior to the laboratory analyses, all samples were thawed and re-examined for aberrations. Upon thawing, they were first rinsed with tap water and then with deionized water in order to remove seawater and impurities. The organisms (approximately 20 colonial zooids) were tapped with blotting paper to dry. For the analyses, all tissues of *C. oblonga* were processed in their entirety.

For analyses requiring lyophilized samples, *C. oblonga* was freeze-dried for 72 h in the CoolSafe lyophilizer (55-9 PRO model, Labogene, Lillerød, Denmark) and pulverized in the vibrational cryogenic mill (SPEX SamplePrep Freezer/Mill 6875, Antylia Scientific, Vernon Hills, IL, USA).

Furthermore, all analyses were conducted in triplicates. Three repetitions were run to ascertain the experimental quality, and good data reproducibility was achieved.

### 2.3. Thermogravimetric Analysis (TGA)

TGA analysis was conducted on the lyophilized *C. oblonga* powder. TGA analyses were performed using SDT Q600 (TA Instruments, New Castle, DE, USA). In each analysis, the sample was placed in an appropriate crucible and subjected to a heating scan from 25 °C to 1000 °C, at a heating rate of 10 °C/min, using 30 mL/min of nitrogen (N_2_) as a carrier gas.

### 2.4. Fourier Transform Infrared (FT-IR) Analysis

The FT-IR spectra of samples were recorded on a FT/IR 4200 spectrophotometer (Jasco Inc., Easton, MD, USA) in an Attenuated Total Reflectance (ATR) mode with zinc selenide (ZnSe) crystal as a focusing component in a wavenumber range 4000–550 cm^−1^, using 32 scans at a 4 cm^−1^ resolution and a 1 cm^−1^ interval at room temperature. Before FT-IR data collection, a background scan was performed for baseline correction.

### 2.5. Proximate Composition

Moisture (water) content was determined gravimetrically after drying to constant weight at 103 ± 2 °C as recommended by AOAC’s Official Methods [27]. Mineral content (ash) was determined by combusting 5 g of sample at 550 °C and weighing the ash after cooling [26]. Crude protein was analysed using the Kjeldahl method and calculated from nitrogen content using the conversion factor N × 6.25 [27]. Crude fat content was determined by the two-step extraction with cyclohexane and propan-2-ol mixtures as solvents [27]. After extraction, solvents were evaporated under vacuum, and the extracted lipids were dried for 3 h at 103 ± 2 °C and weighed.

### 2.6. Fatty Acid Composition

Lipids for determination of fatty acid composition were extracted according to Smedes [28]. To preserve unsaturated fatty acids from oxidation, the final step of the Smedes method, i.e., drying at 103 °C, was excluded from the protocol. Fatty acid methyl esters were prepared by transesterification with methanol according to ISO 5509:2000 method [29].

Gas chromatography was conducted on the Agilent Technologies 6890N Network GC system (Santa Clara, CA, USA) equipped with flame ionization detector. Fatty acid methyl esters (FAMEs) were separated using a DB-23 capillary column (Supplementary Information S1).

Saturated fatty acids (SFA) were calculated as the sum of C:14, C:15, C:16, C:17, C:18, C:20, and C:22. Monounsaturated fatty acids (MUFA) were calculated as the sum of C16:1, C18:1 trans, and C18:1 cis. Polyunsaturated fatty acids (PUFA) were calculated as the sum of C18:2 cis, C18:3n3, C20:4n6, and C22:6n3. Unsaturated fatty acids (UFA) were calculated as the sum of MUFA and PUFA. ω-3 fatty acids were calculated as the sum of C18:3n3 and C22:6n3. The ω-6 fatty acids were calculated as the sum of C20:4n6 and C18:2 cis.

### 2.7. Trace and Macro Elements

Multielement analysis was performed by a triple quadrupole inductively coupled plasma mass spectrometer (ICP-QQQ, 8900, Agilent Technologies Inc., Santa Clara, CA, USA) (Supplementary Information S2). All prepared solutions of lyophilized *C. oblonga* were analysed for total concentration of 32 trace elements (Ag, Al, As, Ba, Be, Bi, Cd, Co, Cr, Cs, Cu, Fe, Li, Mn, Mo, Nb, Ni, Pb, Rb, Sb, Sc, Se, Sn, Sr, Th, Ti, Tl, U, V, W, Y, and Zn) and 6 macroelements (Ca, K, Mg, Na, P, and S). Indium (In, 1 mg/L) was used as an internal standard. Quality control of the analytical procedures used for element analysis was performed by simultaneous analysis of the blank and certified reference material Mussels (NCS ZC 78005, also known as GBW-08571, China National Analysis Centre for Iron and Steel, Beijing, China).

## 3. Results

### 3.1. Temporal Dynamics and Biological Observations

The occurrence and intensity of *C. oblonga* fouling in mussel farms in Lim Bay were investigated from January to December 2023. Based on previous studies, it is known that the colonial corpus usually diminishes after overwintering, depending on local environmental conditions, and initiates vegetative reproduction by forming new zooids from February to April.

**Figure 2:**
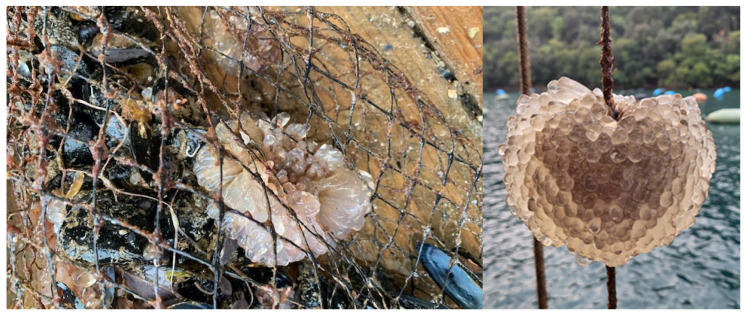
*Clavelina oblonga* on a culture rope with mussels (*Mytilus galloprovincialis*) and infrastructure ropes.

Surprisingly, the biomass of *C. oblonga* was unusually low during the April–May period at the studied sites in Lim Bay (<0.3 kg/40 kg mussel culture ropes). Monthly monitoring of two mussel growing sites (sites S-3 and S-4, Figure 1), in conjunction with communication with the owner of the mussel farm (Istrida d.o.o.), provided information about the first appearance of new colonial zooids in the entire mariculture area in Lim Bay in August. By October, the mussel ropes in the surveyed mariculture farm were heavily fouled with *C. oblonga*, reaching its maximum annual biomass (5–42 kg/40 kg mussel culture ropes, depending on the site and period) (Figure 2).

Slightly higher oxygen concentrations and salinity were recorded on sites with pronounced *C. oblonga* presence (S-3 and S-4). The temperature remained quite uniform throughout the sampled area on the sampled date (October 2023), indicating that the water column was not stratified during profiling (Supplementary Figure S2a–e).

### 3.2. Thermal Properties

**Figure 3:**
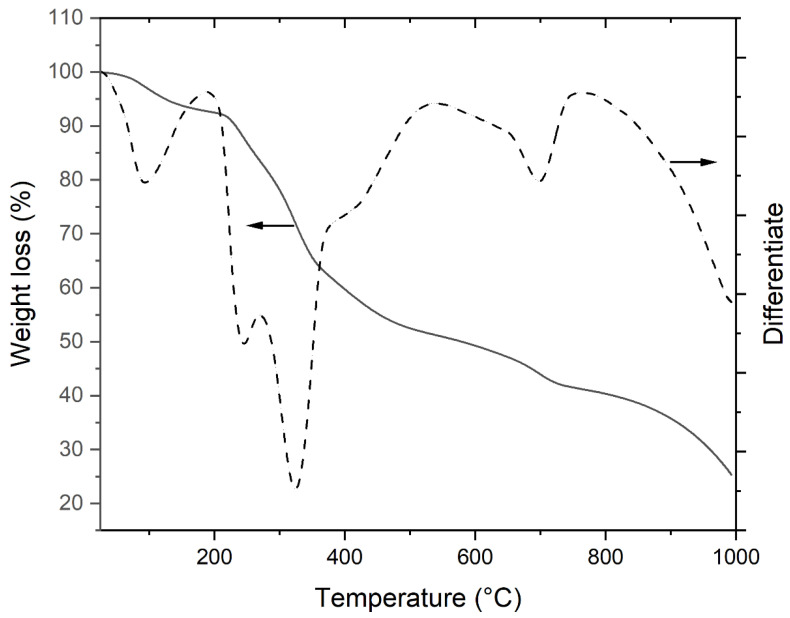
TGA and DTG curves of *Clavelina oblonga*.

Figure 3 shows the TGA and DTG curves of the dried *C. oblonga* mass at a heating rate of 10 °C min^−1^, with the pyrolytic characteristics in an inert or N_2_ environment, where the heating temperature ranged from 25 °C to 1000 °C.

Analysis of the DTG curves clearly distinguishes five peaks: one at 97 °C that confirms the dehydration and loss of volatile compounds, the two main peaks at 245 °C and at 323 °C due to degradation of carbohydrates and proteins, a slight shoulder at 420 °C due to lipid degradation, and the peaks at 697 °C, corresponding, respectively, to the carbonaceous compounds and residual ash (Figure 3).

### 3.3. Fourier Transform Infrared (FT-IR) Analysis

**Figure 4:**
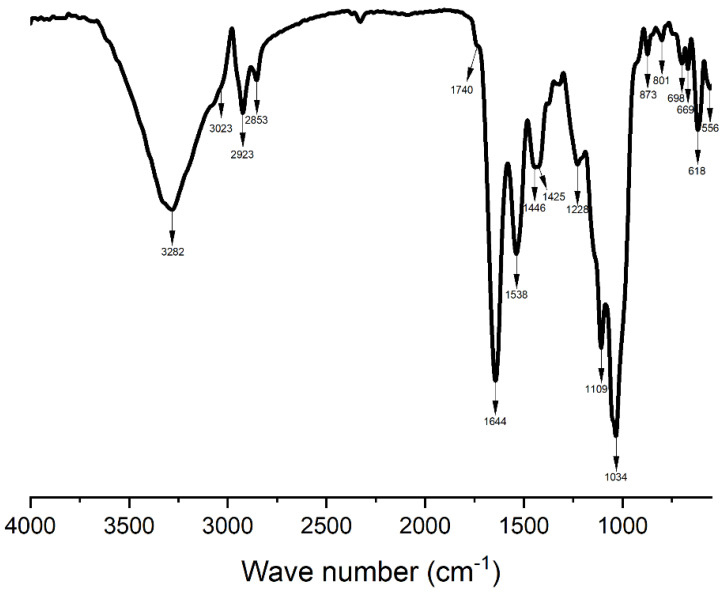
Fourier Transform Infrared (FT-IR) analysis of the *Clavelina oblonga* composite structure.

The composite structure of *C. oblonga* was further verified by FT-IR (Figure 4). The spectrum exhibited several strong peaks to be considered for further examination and attribution. The first one was a very broad band at 3282 cm^−1^ belonging to both O-H and N-H stretching of cellulose and protein, respectively. The narrow peaks at 2923 and 2853 cm^−1^ refer to alkyl C-H bonds. More intense peaks were recorded at 1644 and 1538 cm^−1^ belonging to proteins [30], while the very strong signal at 1034 cm^−1^ is related to C-O-C stretching of cellulose. The presence of C=O bonds of lipid esters is confirmed by the low intensity peak at 1740 cm^−1^. There were different small and narrow signals, like the ones recorded at 873 and 1228 cm^−1^, that contributed to the FT-IR spectral profile of *C. oblonga* and were found also in the IR spectrum of tunichrome, an important peptide contained in many tunicate species [31,32].

### 3.4. Proximate Composition

All samples primarily consisted of water (moisture) as the major constituent in their proximate body composition (Table 1). Despite rinsing the free water and drying the organisms using paper towels, there is a possibility of minimal residual water within the inner body tissues during analysis. *C. oblonga* accordingly exhibited a moisture content of 95.44%. The organic components, constituting proteins and lipids, were expressed on a dry basis.

### 3.5. Fatty Acid Composition

**Figure 5:**
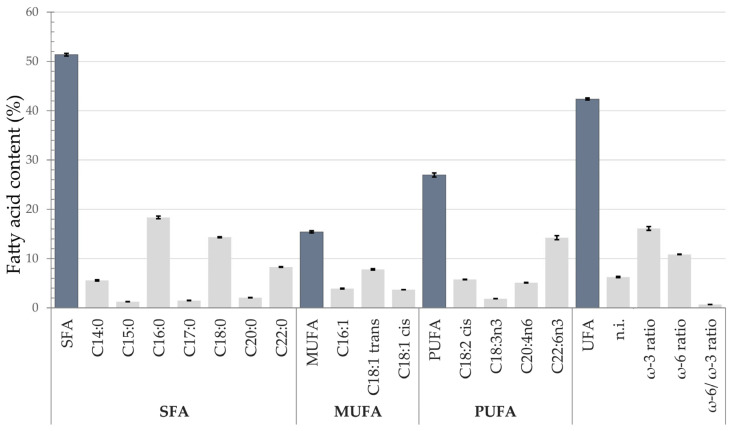
Fatty acid composition of *Clavelina oblonga* collected from the mussel farming site in the northern Adriatic Sea, Croatia. Abbreviations: SFA—saturated fatty acids, MUFA—monounsaturated fatty acids, PUFA—polyunsaturated fatty acids, UFA—unsaturated fatty acids, n.i.—nonidentified fatty acids.

The major fatty acids in the entire tissues of *C. oblonga* (Supplementary Table S1) were palmitic acid, PA (C16:0), stearic acid, SA (C18:0), and docosahexaenoic acid, DHA (C22:6n3), followed by docosanoic acid, DA (C22:0), elaidic acid, EA (C18:1 trans), linoleic acid, LA (C18:2 cis), and myristic acid, MA (C14:0) (Figure 5). Fatty acid composition is expressed as a percentage of total fatty acids, representing SFA, MUFA, PUFA, UFA, ω-3 fatty acids, and ω-6 fatty acids (Figure 5). The three groups of fatty acids (SFA, MUFA, PUFA) accounted for 51.37%, 15.41%, and 26.96% of total fatty acids, respectively. The total UFA accounted for 42.37%. The ω-3 PUFA ratio was 16.10%, ω-6 PUFA ratio was 10.86%, while ω-6/ω-3 ratio amounted to 0.68. The nonidentified fatty acids comprised 6.26%.

### 3.6. Trace and Macroelements

A total of 32 trace elements and 6 macroelements were measured in entire *C. oblonga* tissues (Supplementary Table S2). Of trace elements, Al was predominant, the sequence of concentration (in µg/g DW) being Al (1843) > Fe (1274) > Sr (140) > Ti (113) > Mn (50.8) > V (37.6) > Zn (35.8) > Cu (19.8) > Ba (8.87) > As (7.18) > Li (5.73) > Rb (5.72) > Ni (4.67) > Cr (4.40) > Pb (2.15) > Mo (1.46) > Se (1.36) > Co (0.82) > Y (0.60) > Nb (0.47) > Th (0.44) > Sc (0.43) > Cs (0.33) > Sn (0.27) > Ag (0.21) > U (0.21) > W (0.20) > Be (0.12) > Cd (0.11) > Sb (0.06) > Tl (0.05) > Bi (0.03).

Among the analysed macroelements, Na was predominant, the sequence of concentration (in mg/g DW) being Na (99.1) > S (29.2) > Mg (17.6) > Ca (11.1) > K (5.21) > P (2.06).

## 4. Discussion

The studied area of the northern Adriatic experiences a diverse range of temperatures, following a distinct annual cycle, with daily fluctuations influenced by tides, winds, and currents. Seasonal average values vary from approximately 11–14 °C in winter (March) to 19–27 °C in summer (August), as previously outlined [24]. Additionally, this mariculture site is affected by freshwater, particularly during rainy days in spring and fall, when small streams and springs flow into the coved part of Lim Bay [24]. The coved section of Lim Bay (mariculture site S-1) exhibited more significant salinity fluctuations (8–37 psu), while the middle part (mariculture site S-4) demonstrated minor variations in surface salinity (33–37 psu), mirroring the entrance of the bay into the open sea. The influx of freshwater in the channel’s coved part was associated with elevated terrigenous nutrient uptake. Consequently, the increased dissolved organic matter contributed to a rise in planktonic mass, positively influencing mussel growth rates [25], but also creating favourable conditions for *C. oblonga* expansion. Given that fluctuations in environmental conditions, such as reduced salinity, can trigger diverse cellular responses and adaptations in marine organisms [25,36,37], it has been demonstrated that hypoosmotic stress (S < 20 psu) can serve as an effective measure to prevent the intense overgrowth/biofouling of *C. oblonga* on mussels in mariculture [13].

The ascidian tunicate species *C. oblonga* remains surprisingly understudied in the Adriatic Sea and throughout the Mediterranean, with a notable absence of comparable data. Consequently, when drawing parallels between its biological and biochemical traits and those of other tunicates, we primarily correlated them with the characteristics of other ascidians that may also be found in the Mediterranean region.

To that end, the ash content of *C. oblonga* (29.1%), the second most abundant inorganic component after moisture, was notably lower than that of other ascidian species from the northern Adriatic Sea, which ranged from 44.1% to 65.6%, depending on the species [35]. The ash content was higher in other tunicate species and accounted for 56% of dry weight of *Salpa thompsoni* [38]. As ash content represents the inorganic mineral components left after combustion, it serves as an indicator of the sample’s mineral composition, which may vary based on sea salinity. In that sense, the high ash content observed in the inner body tissues of the ascidians could stem from the elevated salinity of the sea [39]. The lower sea salinity in Lim Bay, owing to freshwater inflow, fosters ideal conditions for mussel farming [40], and seemingly contributes to the reduced ash content in *C. oblonga*. When comparing with results from other regions, it should be considered that *C. oblonga* may retain variable amounts of seawater, either of higher or lower salinity, within its body, which could have contributed to the observed differences in ash values [41].

In the pyrolytic process with temperature range of 450–600 °C, the tunicate mass decayed slightly, indicating the decomposition of lipids, which usually occurs at higher temperatures with respect to proteins and carbohydrates [42]. The FT-IR analysis confirmed the presence of lipids [43,44], proteins [45], and cellulose in *C. oblonga* [46,47]. In addition, FT-IR spectrum of *C. oblonga* also shows peaks typical of tunichrome, an important antioxidant and antibacterial compound found in other tunicate species [30,31], confirming that *C. oblonga* is a source of high-quality substances, as in all tunicates [1]. Interestingly, among the organic constituents, the protein content of *C. oblonga* on a dry basis (39.2%) had similar value when compared to other ascidian species from the Adriatic Sea, which ranged from 26.0 to 41.9% [35]. Protein content, a crucial component of tunicates contributing to their overall nutritional value, exhibits variation among species growing in similar environmental conditions. The protein content observed in *C. oblonga* may play an adaptive role and contribute to the invasiveness of this species. Previous studies suggest that certain proteins favour the diversity of tunicate-associated microbiota, which is essential for protecting them from pathogens and strengthening their immune system [48]. In addition, lipid content in *C. oblonga* (8.6%) was notably higher compared to other ascidians present in the Adriatic Sea, which ranged from 2.4 to 4.0% [35]. The northern Adriatic experiences substantial thermal variations during different seasons, prompting ascidians to increase their lipid content in colder months, consequently storing higher energy amounts [49]. It is also significantly impacted by freshwater runoff from the Po River. Consequently, during fall, there is an increased abundance of phytoplankton and chlorophyll maxima in the oligotrophic north-eastern part of the Adriatic Sea [44,50]. As previously reported for other tunicates [51], changes in temperature or salinity may contribute to the observed increase of the overall *C. oblonga* lipid contents. The combined quantities of proteins, lipids, ash, and calculated carbohydrates roughly account for over two-thirds of the dry weight of *C. oblonga*, similar to the findings regarding the sum of *S. thompsoni*’s dry weight components [38]. Given the high rate of organic compounds observed in *C. oblonga*, further research is needed to assess the potential of this tunicate species as a source of bioactive compounds for developing novel pharmaceuticals.

Upon comparing the limited available data on fatty acids in tunicates, it can be concluded that the fatty acid composition of *C. oblonga* differs from that of other tunicates. Clearly, diet influences the chemical and biochemical composition of tunicates. Although there is a slight uncertainty as to what exactly constitutes the diet of ascidians, their food is restricted by size and mainly consists of phytoplankton and marine bacteria [52]. Phytoplankton actively biosynthesize fatty acids of a shorter chain. Thus, phytoplankton often accumulate MA, as well as PUFA of the C16, C18, and C20 series. In zooplankton, MA is a minor constituent, and the major PUFA are in the C20 and C22 series [53]. Nevertheless, the filter-feeding *C. oblonga* seems to possess a high proportion of C16:0, despite retaining a smaller amount of MA.

The findings on fatty acids in *C. oblonga* only partially align with the fatty acid composition of *Ascidia* sp. [39]. Namely, the fatty acid analysis of the entire tissues of *C. oblonga* demonstrated that SFA were found to be the most abundant, followed by unsaturated fatty acids, among which MUFA were the least prevalent. While *Ascidia* sp. displayed similar SFA and MUFA compositions in its tunics, it exhibited slightly higher levels of PUFA compared to MUFA in its inner body tissues [39]. The prevalence of individual fatty acids differs between *Ascidia* sp. and *C. oblonga*. In both tissues of *Ascidia* sp., oleic acid, OA (C18:1) was the most abundant, contrasting with palmitic acid (PA) in *C. oblonga*. Indeed, within the tunicates, only *Thalia democratica* also displayed the highest content of PA among all fatty acids. This content varied depending on the sampling location, with a prevalence of DHA in colder locations [33]. Conversely, in the ascidian *S. plicata*, eicosapentaenoic acid, EPA (C20:5) was the most prevalent, a component not even measurable in *C. oblonga* [39]. Despite this disparity, both organisms exhibited a dominance of ω-3 fatty acids within omega fatty acids, although their ratios greatly exceeded those found in *C. oblonga* [1]. Interestingly, tunicates of the East Sea (sea squirt, *Halocynthia aurantium*) in general had high prevalence of EPA and DHA, while PA was the least abundant [54]. Somewhat similar to the findings in *C. oblonga*, *H. aurantium* exhibited SFA as the predominant lipid category (63.75%), followed by PUFA at 19.34% and MUFA at 16.92% [55]. The chemical and fatty acid composition of *C. oblonga* indicates that it could be a valuable source of nutrients. Its nutritional profile resembles that of several seafood products [1], positioning it as a viable option for both food and feed uses. Our findings align with previous reports that marine-derived lipids have more complex chemical structures than lipids derived from other sources of fats or oils, primary due to their highly diverse fatty acid composition [56]. However, tunicates exhibit higher levels of SFA compared to some marine sources present in the Mediterranean diet, such as mussels and fish, which are characterized by a fatty acid profile more favourable for human consumption [41,57]. For example, mussels (*Mytilus galloprovincialis*) and fillets of gilthead seabream (*Sparus aurata*) contain approximately 30% SFA, representing half the amount of SFA found in *C. oblonga*. Although the differences in UFA downregulation and lipid accumulation appear somewhat contrasting, they may reflect specific metabolic responses to environmental conditions and food availability that require further investigation. Despite the observed differences, it should be noted that the potential utilization of *C. oblonga*, either for human consumption (food) or for the extraction of valuable bioactive compounds, offers a sustainable approach to repurposing an invasive and biofouling marine organism.

The concentration of trace metals in all marine invertebrates exceeds that found in seawater, whether these elements are essential or not [58]. This trend is true also for *C. oblonga*, as indicated by concentration levels found in the northern Adriatic Sea [59]. The northern Adriatic, in the proximity of the sampling point for *C. oblonga*, exhibited dissolved trace metal concentrations in the sequence Zn > Ni > Cu > Co > Pb > Cd [59], partially corresponding to their order in *C. oblonga* (Zn > Cu > Ni > Pb > Co > Cd). Similarly to *C. oblonga*, Zn concentrations in seawater were the highest, surpassing those found throughout the Mediterranean, with a tendency to decrease in fall [59]. However, recent studies on *M. galloprovincialis* have shown higher Zn accumulation in mussel tissue during the fall compared to spring and summer, with the highest levels observed in winter (76.2–167.5 µg/g DW) [60]. Concerning toxic metals, *C. oblonga* exhibited lower concentrations of As (7.18 vs. 18.2–43.2 µg/g) and higher of Pb (2.15 vs. 0.7–1.86 µg/g) than the mussels in the Istria region that it fouls [60]. The levels of As in *C. oblonga* were lower than in the ascidians *C. intestinalis* or *Ascidia* sp. [39] and could be both species-specific and related to temporary elemental variations in the environment. The European Commission Regulation 2023/915 on the maximum levels for certain contaminants in food does not list As for the seafood products. However, the Pb concentrations in bivalve molluscs are limited to 1.50 µg/g. The obtained Pb concentrations in *C. oblonga* were higher than those in mussels from the same area (2.15 vs. 0.7–1.86 µg/g) [60], but lower than Pb concentrations in mussels grown in the further north of the Adriatic Sea (11.5 µg/g) [61,62]. Being efficient filter feeders, ascidians can accumulate and concentrate trace metals deriving from seawater and their food intake, including Cd, Cr, Fe, Mg, Mo, Nb, Ta, Ti, and V [63,64]. Essential trace elements such as Cu, Fe, Mn, and Zn play crucial roles as components of their enzymes and electron transport systems. The preferential accumulation site of essential trace elements is the branchial basket, as vital functions of ascidians, such as breeding, absorption, storage, and circulation, take place there [65]. Conversely, Al, As, Cd, Hg, Ni, Pb, and V do not possess a biological role in marine invertebrates and may instead be toxic [58,63]. A hyperaccumulation of V from seawater via vanadocytes was previously demonstrated in tunicates [1]. This study found a 34-fold higher concentration of Fe than V, correlating with their relationship in *S. plicata* [65]. This finding might suggest that ascidians accumulate Fe rather than V, although V accumulation, particularly in tunic, might serve against predation, making them unpalatable to predators [65]. Differences in elemental composition may result from the presence of either upregulated or species-specific metallothioneins involved in both metal transport and stress protection mechanisms [66]. Such changes in biochemical composition may be related to the invasiveness processes, as invasive species generally tend to develop different traits in non-native areas. This adaptation results in variations in the species-specific biochemical composition and potentially depends upon habitat invasion and settlement. Further analyses should be conducted in other environmental conditions, with longer or shorter invasion history, where *C. oblonga* poses a significant threat. Therefore, the concentration of analysed elements should be further compared with regional specimens of *C. oblonga*, considering that the total concentration of individual elements does not always reflect their bioavailability through food consumption [61]. Lastly, as demonstrated in this study, the accumulation and concentration of toxic and trace metals must be carefully evaluated when considering the potential use of *C. oblonga* biomass for feed and food purposes.

## 5. Conclusions

The climate change and human-mediated redistribution of species have led to the establishment of exotic invasive populations of ascidians, such as *C. oblonga*. The increase of its occurrence in the northern Adriatic Sea is a result of changes in the abiotic conditions of the sea, particularly its warming, and other processes contributing to the expansion area of *C. oblonga*. There is a strong possibility of the permanent establishment of *C. oblonga* in the Adriatic Sea, with inconceivable impacts on ecosystem structure, mussel farming, plankton biomass, and the distribution of other marine species. *C. oblonga* has seemingly adapted to the environmental conditions in the Adriatic Sea, reaching its maximum spread and biomass from late summer to mid-autumn. Due to the sea temperatures recorded in the Istrian mussel farming areas, further spread in the northern Adriatic is anticipated in the future. Yet, its occurrence in natural habitats outside aquaculture areas was not observed in the region. *C. oblonga* is thus a strong competitor in the northern Adriatic due to its rapid growth rate, pronounced seasonal recruitment, and ability to recover from physical removal or predatory damage. Abiotic factors in the marine environment are likely to enhance or limit its dominance more than biotic interactions, although its generally high protein content may serve an adaptive function and contribute to the species’ invasiveness.

By collaborating with local mussel farmers, investigating and proposing eradication measures with the aim of potentially mitigating the threat and reducing this tunicate invasion, we can recommend, based on the current state of knowledge, avoiding the handling of mussels during the sexual reproduction of *C. oblonga*. Additionally, the removal of this fouling species at the end of the fall is suggested to reduce the chances of overwintering and the appearance of new zooids through vegetative reproduction at the end of the winter season. In this context, *C. oblonga* could be regarded as an additional source of biomass that could be further adopted as a sustainable source of valuable compounds.

The results reported here offer first information on the proximal, fatty acid, macroelement, trace element, and toxic metal composition of *C. oblonga* collected at the mussel farming site in Lim Bay of the northern Adriatic. Even if there is limited knowledge on the potential use of tunicate ascidians grown in the Mediterranean region, studies conducted in other areas highlight the potential use of this group of organisms in animal and human food consumption and discovery of novel bioactive compounds. While some organic compounds were undetected in this study, the derived nutritional values of *C. oblonga* should not be disregarded. It should be considered that the herewith presented results highlight the potential of adopting invasive species to extract non-targeted resources, offering a promising approach to reduce the carbon footprint associated with the production of other high-protein foods. The results of this study also suggest that *C. oblonga* could potentially serve as a sustainable protein source to replace fishmeal, offering both nutritional value and acting as an attractant for fish. In addition, its high content of cellulose renders this invasive species an appealing feedstock of high-value products, such as nanocellulose. Further studies are needed to determine how *C. oblonga* will impact the marine environment and species diversity in the future, as well as to identify the singularity of its bioactive compounds for potential development of functional products.

In addition, to reduce the dispersal of *C. oblonga*, several measures must be implemented, including ballast water management, regular biofouling control on aquaculture gear and boat hulls, frequent surveys in aquaculture areas, increased public awareness, and the introduction of best practices to prevent its spread.

## Figures and Tables

**Table 1:** Comparison of proximate composition (% dry weight) of *Clavelina oblonga* with literature data.

Analyte (%)	*Clavelina oblonga*	*Cnemidocarpa verrucosa*	*Polycitor adriaticus*	*Aplidium conicum*	*Aplidium elegans*	*Styela plicata*	*Botryllus schlosseri*	*Botrylloides violaceus*
Moisture	95.440 ± 0.003	-	-	-	-	-	-	-
Ash	29.1 ± 1.7	38.8 ± 1.7	44.1 ± 2.4	65.6 ± 5.7	52.7 ± 1.8	46.2 ± 8.6	55.4 ± 8.1	52.9 ± 0.6
Proteins	39.2 ± 0.7	55.3 ± 1.4	41.9 ± 4.0	26.0 ± 2.9	37.3 ± 4.3	40.9 ± 13.1	38.2 ± 7.4	41.5 ± 2.4
Lipids	8.6 ± 0.5	4.9 ± 0.9	3.2 ± 1.1	3.2 ± 1.3	4.0 ± 0.3	3.2 ± 0.8	2.9 ± 0.5	2.4 ± 0.4

Colonies were collected from the mussel farming site in the Northern Adriatic Sea, Croatia. The results are compared with literature data for other ascidian species: *Cnemidocarpa verrucosa* (Antarctica) [33,34], *Polycitor adriaticus*, *Aplidium conicum*, *Aplidium elegans*, *Styela plicata*, *Botryllus schlosseri,* and *Botrylloides violaceus* (Adriatic Sea) [35]. Reported values represent the sum of soluble and insoluble proteins. The results are presented as mean estimates ± standard error of the mean, where accessible.

## Data Availability

Dataset available on request from the authors.

## Supplementary Materials

The following supporting information can be downloaded at: https://www.mdpi.com/article/10.3390/ani15101371/s1, Supplementary Information S1: Fatty acid composition; Supplementary Information S2: Trace and macro elements; Supplementary Figure S1: *Clavelina oblonga* on culture ropes with mussels (*Mytilus galloprovincialis*) (a,b) and infrastructure ropes (c–e), sampled specimens of *C. oblonga* (f); Supplementary Figure S2: Seawater column characteristics (temperature, conductivity/salinity, dissolved oxygen and fluorescence/chlorophyll-a) during *Clavelina oblonga* sampling at mariculture area in Lim Bay (October 2023), and fluctuations in average sea temperatures at a target depth of 5 m (S-3) (2021–2023); Supplementary Table S1: Fatty acid composition, expressed as a percentage of the total fatty acids, analysed in the entire tissues of *Clavelina oblonga* collected from the mussel farming site in the northern Adriatic Sea, Croatia; Supplementary Table S2: Total concentration of trace elements and macroelements in entire tissues of *Clavelina oblonga*; Supplementary Video S1: Biofouling of mussel culture ropes with *Clavelina oblonga.*
