# Supplementary material for: Biological, Biochemical and Elemental Traits of Clavelina oblonga, an Invasive Tunicate in the Adriatic Sea
Source: Animals (Basel). 2025 May 9;15(10):1371. doi: 10.3390/ani15101371 (PMC12108202; doi:10.3390/ani15101371)
Supplement: Supplementary file 1 [file animals-15-01371-s001.zip › Supplementary materials.pdf]

Supplementary material
Biological, biochemical and elemental traits of *Clavelina oblonga*, an invasive tunicate in the Adriatic Sea

Natalija Topić Popović, Bojan Hamer, Ivančica Strunjak-Perović, Tibor Janči, Željka Fiket, Matilda Mali, Luca Privileggio, Kristina Grozić, Dijana Pavičić-Hamer, Lucija Vranjković, Tamara Vujović, Marija Miloš, Maria Michela Dell'Anna, Darya Nefedova and Rozelindra Čož-Rakovac

Materials and methods

Supplementary information S1. Fatty acid composition

For the analysis of fatty acids, 60 mg of extracted lipids was dissolved in 4 mL of isooctane and 200 μL of potassium hydroxide in methanol (2 mol/L) were added. The mixture was vortexed for 30 s and left for a few minutes at room temperature to react. Afterwards, 1 g of sodium hydrogen sulphate monohydrate was added, mixed, and clear supernatant containing methyl esters was transferred into the vial.

Gas chromatographic analysis was conducted on the Agilent Technologies 6890N Network GC system (USA) equipped with flame ionization detector. Helium was used as a carrier gas with a constant flow of 1.5 mL/min. The injector temperature was set at 250 °C and detector at 280 °C. The oven temperature was programmed to increase by 7 °C/min from initial 60 °C to final temperature of 220 °C where it was held for 17 min. The split ratio was 30:1. Fatty acids were identified by comparing their retention times with the retention times of 37 Component FAME Mix (Supelco, Sigma-Aldrich, USA). The surface normalization method was used to determine the quantitative composition of fatty acids expressed as the percentage of total fatty acids.

Supplementary information S2. Trace and macro elements

Prior to analysis, subsamples (0.1 g) of lyophilized tunicates were subjected to a total digestion in the microwave oven (Multiwave ECO, Anton Paar, Austria) in a one-step procedure consisting of digestion with a mixture of 6 mL nitric acid (HNO_3) and 0.1 mL hydrofluoric acid. After digestion, samples were acidified with 2% (v/v) HNO_3 without further dilution.

Supplementary Figures and Tables

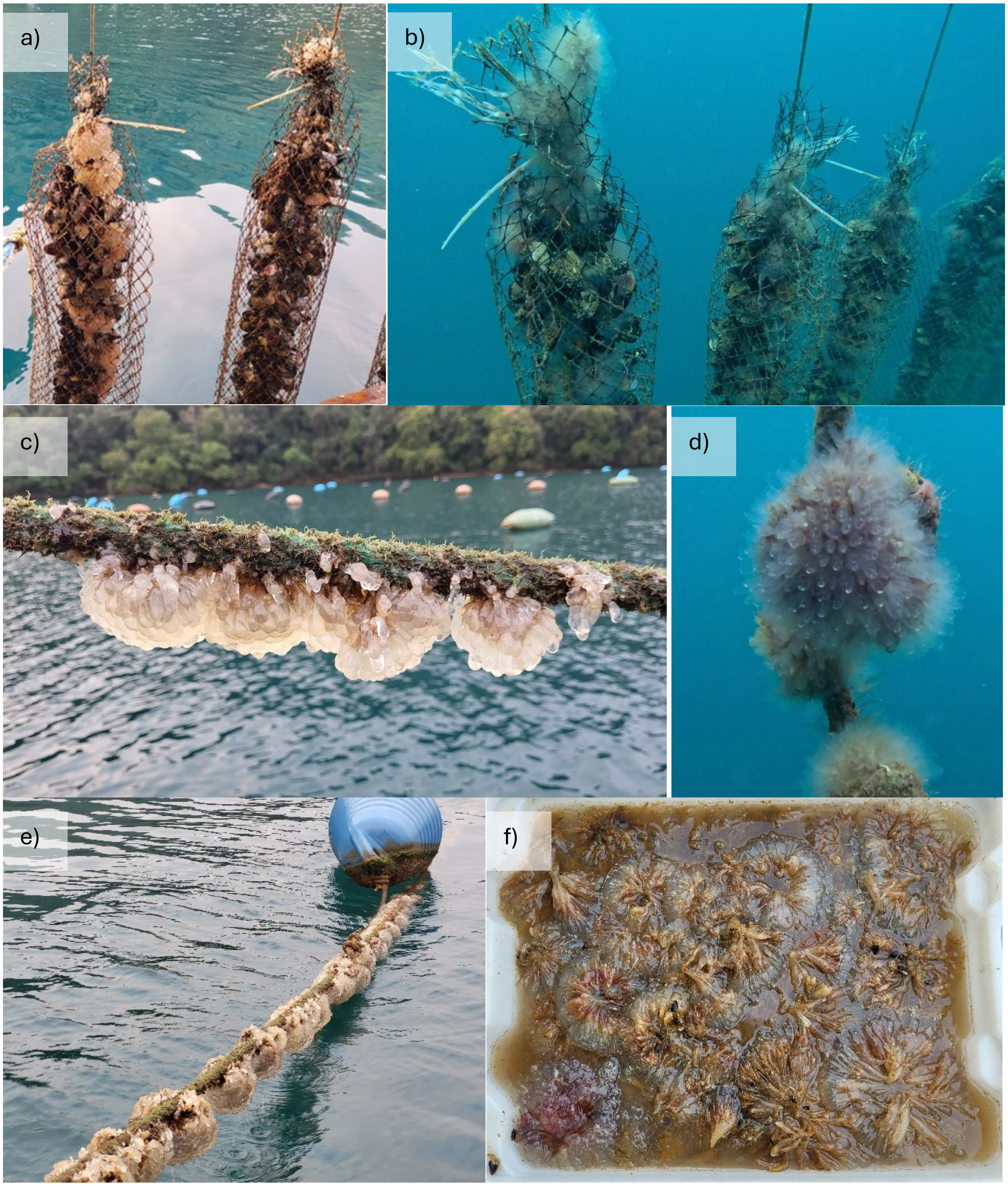

Supplementary Figure S1. *Clavelina oblonga* on culture ropes with mussels (*Mytilus galloprovincialis*) (a, b) and infrastructure ropes (c, d, e), sampled specimens of *C. oblonga* (f).

Supplementary Figure S2. Seawater column characteristics (temperature, conductivity/salinity, dissolved oxygen and fluorescence/chlorophyll-a) during *Clavelina oblonga* sampling at mariculture area in Lim Bay (October 2023), and fluctuations in average sea temperatures at a target depth of 5 m (S-3) (2021–2023).

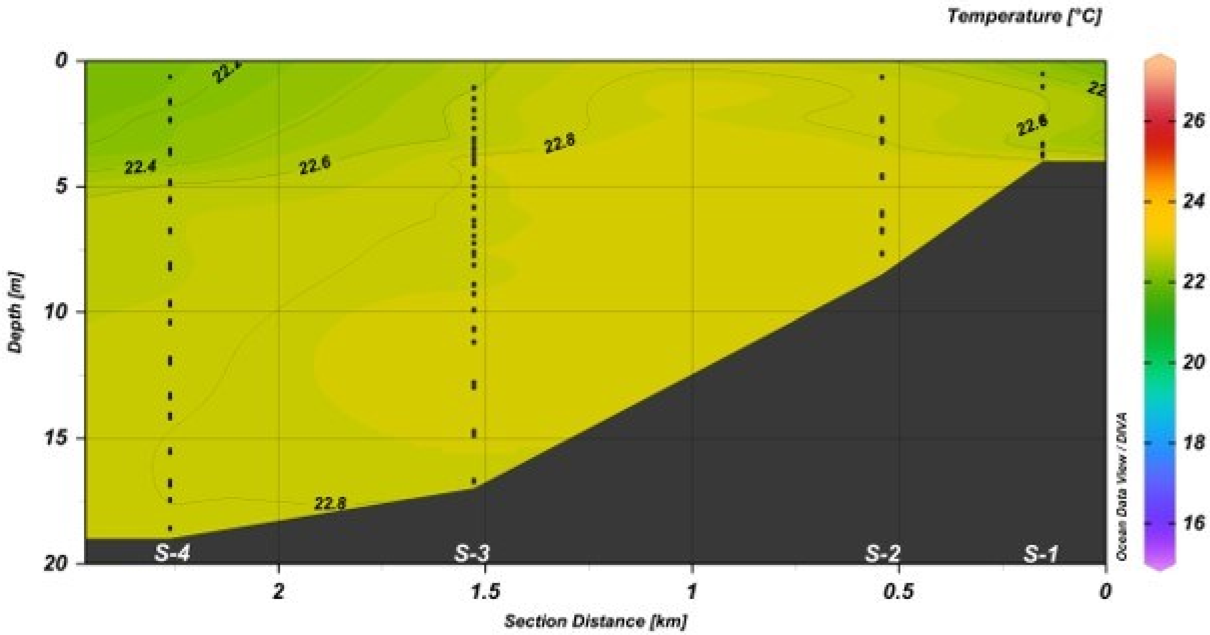

Supplementary Figure S2a. Temperature of seawater (°C)

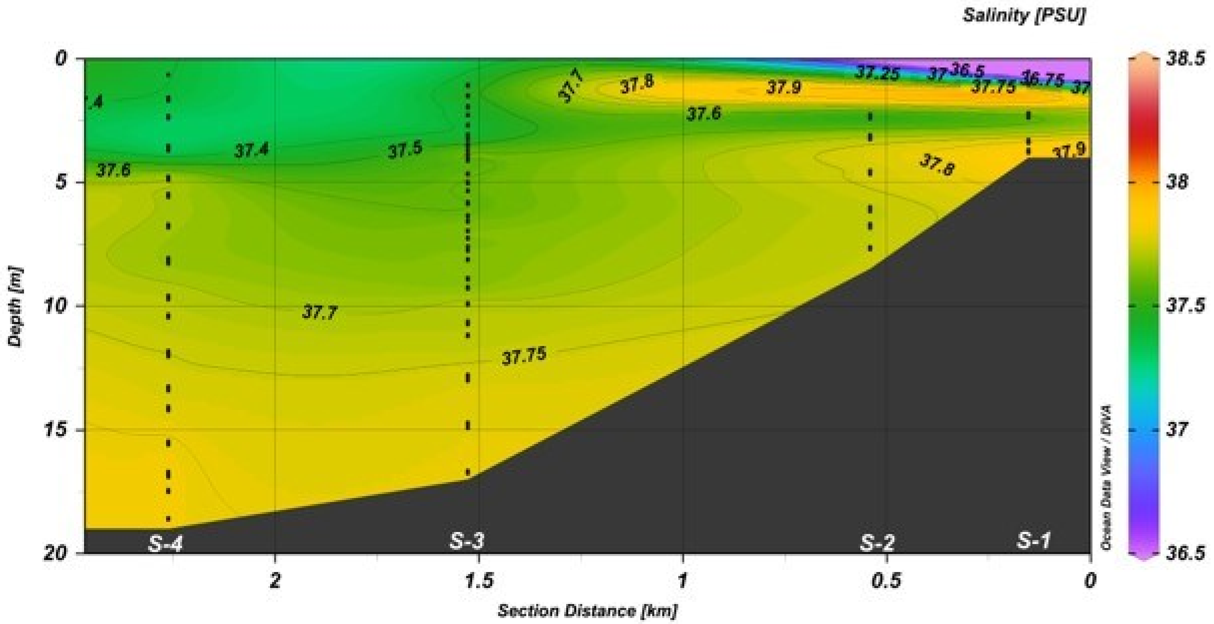

Supplementary Figure S2b. Salinity of seawater (psu)

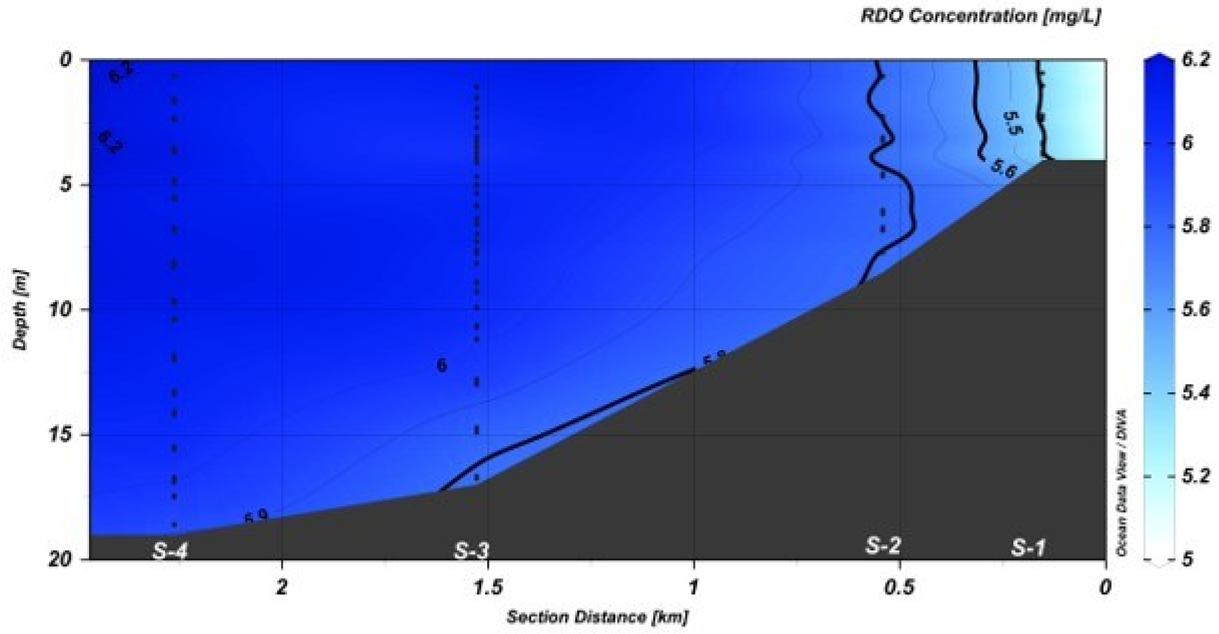

Supplementary Figure S2c. Dissolved oxygen concentration in the seawater (mg/L)

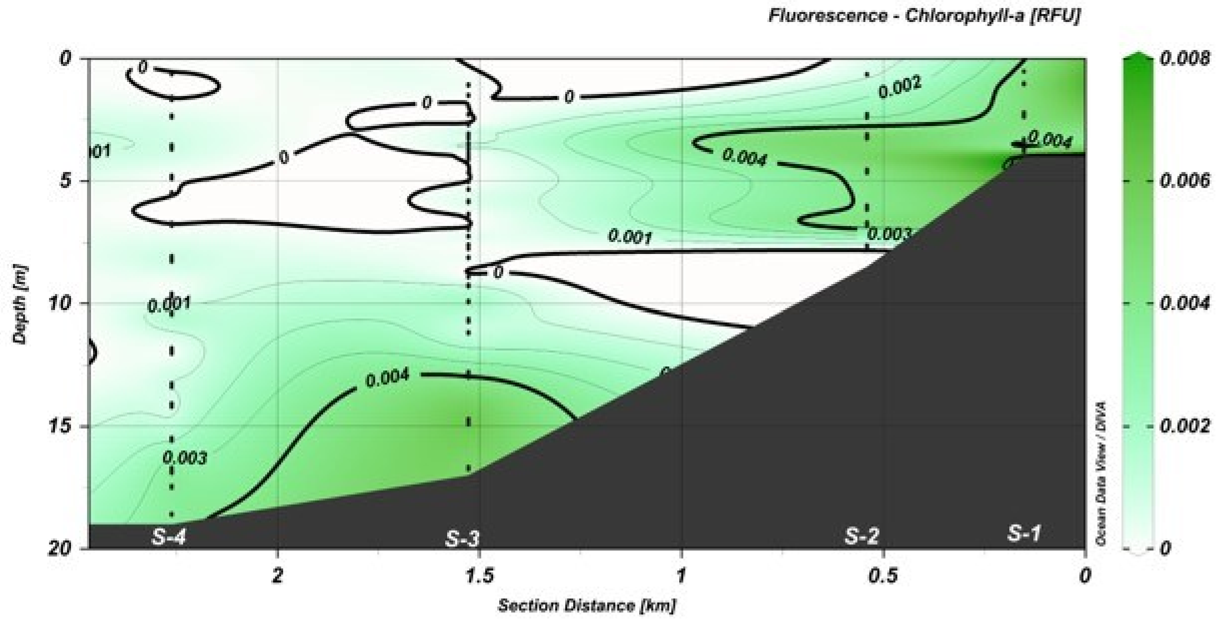

Supplementary Figure S2d. Chlorophyll-a in seawater (relative fluorescence units, RFU)

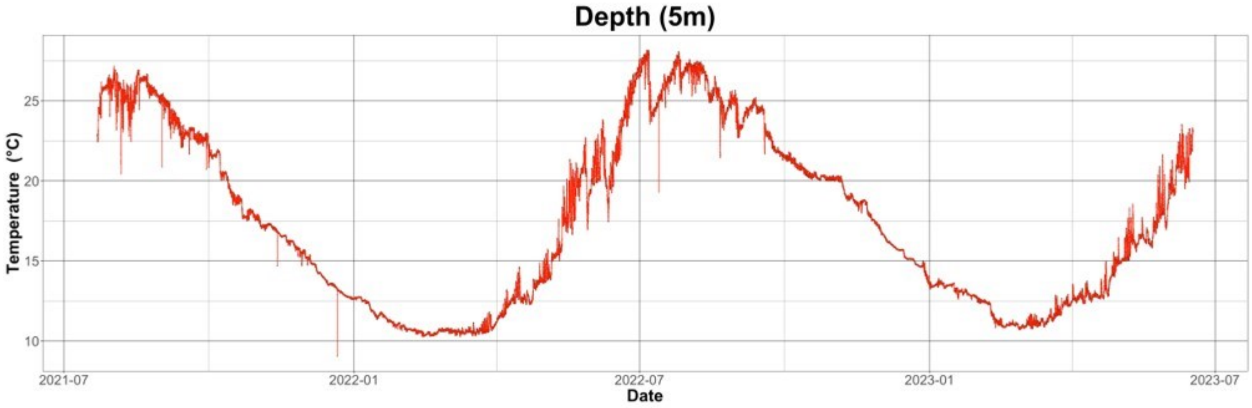

Supplementary Figure S2e. Temperature (°C) of seawater at a depth of 5 m used for mussel growth and biofouling monitoring, measured over a period 2021–2023 using data logger HOBO MX2203 (Onset, USA). In addition, we used a data logger U24-002-C to measure salinity but because of intense biofouling and *Clavelina oblonga* overgrowth of logger sensor the measured values of conductivity were intermittent and unreliable without e.g. Cu protection.

Supplementary Table S1. Fatty acid composition, expressed as a percentage of total fatty acids, analysed in the entire tissues of *Clavelina oblonga* collected from the mussel farming site in the northern Adriatic Sea, Croatia.

Fatty acids	Mean ± standard error
C14:0	5.57 ± 0.14
C15:0	1.27 ± 0.02
C16:0	18.35 ± 0.28
C16:1	3.90 ± 0.09
C17:0	1.49 ± 0.02
C18:0	14.33 ± 0.09
C18:1 trans	7.81 ± 0.13
C18:1 cis	3.69 ± 0.01
C18:2 cis	5.77 ± 0.05
C18:3n3	1.85 ± 0.03
C20:0	2.07 ± 0.04
C20:4n6	5.09 ± 0.06
C22:0	8.28 ± 0.08
C22:6n3	14.24 ± 0.41
n.i.*	6.26 ± 0.12
SFA	51.37 ± 0.27
MUFA	15.41 ± 0.22
PUFA	26.96 ± 0.40
UFA	42.37 ± 0.18
ω-3 ratio	16.10 ± 0.38
ω-6 ratio	10.86 ± 0.04
ω-6/ ω-3 ratio	0.68 ± 0.01

*not identified

Supplementary Table S2. Total concentration of trace elements and macroelements in entire tissues of *Clavelina oblonga*.

Analysed element	*Clavelina oblonga* (n = 6)	*Microcosmus exasperatus*	*Phallusia nigra*	*Ciona intestinalis*	*Microcosmus sulcatus*	*Ascidia* sp.
**Trace elements (µg/g DW)**						
Ag – Silver	0.21 ± 0.05	-	-	0.021	0.031	0.33
Al – Aluminium	1843 ± 676	2763	5697	-	-	898
As – Arsenic	7.18 ± 1.23	-	-	-	-	17.21
Ba – Barium	8.87 ± 2.61	-	-	-	-	-
Be – Beryllium	0.12 ± 0.05	-	-	-	-	-
Bi – Bismuth	0.03 ± 0.01	-	-	-	-	-
Cd – Cadmium	0.11 ± 0.01	0.08	0.1	-	-	0.26
Co – Cobalt	0.82 ± 0.19	2	3	0.52	1.9	-
Cr – Chromium	4.40 ± 1.55	-	-	5.5	6.6	2.55
Cs – Caesium	0.33 ± 0.12	-	-	0.071	0.047	-
Cu – Copper	19.8 ± 4.5	10	29	-	-	21.85
Fe – Iron	1274 ± 467	2564	5217	880	840	1241
Li – Lithium	5.73 ± 1.23	-	-	-	-	2.37
Mn – Manganese	50.8 ± 5.5	79	97	-	-	-
Mo – Molybdenum	1.46 ± 0.25	-	-	-	-	-
Nb – Niobium	0.47 ± 0.17	-	-	-	-	-
Ni – Nickel	4.67 ± 0.95	3	5	-	-	3.48
Pb – Lead	2.15 ± 0.68	4	6	-	-	2.97
Rb – Rubidium	5.72 ± 1.54	-	-	2.6	2.6	-
Sb – Antimony	0.06 ± 0.02	-	-	0.16	0.10	-
Sc – Scandium	0.43 ± 0.16	-	-	0.15	0.078	-
Se – Selenium	1.36 ± 0.20	-	-	1.2	5.1	6.70
Sn – Tin	0.27 ± 0.13	-	-	-	-	0.12
Sr – Strontium	140 ± 9	-	-	-	-	-
Th – Thorium	0.44 ± 0.15	-	-	-	-	-
Ti – Titanium	113 ± 40	-	-	-	-	-
Tl – Thallium	0.05 ± 0.02	-	-	-	-	-
U – Uranium	0.21 ± 0.04	-	-	-	-	-
V – Vanadium	37.6 ± 5.5	112	3426	-	-	1611
W – Tungsten	0.20 ± 0.06	-	-	-	-	-
Y – Yttrium	0.60 ± 0.20	-	-	-	-	-
Zn – Zinc	35.8 ± 4.8	60	39	100	180	42.08
**Macroelements (mg/g DW)**						
Ca – Calcium	11.1 ± 1.6	-	-	-	-	-
K – Potassium	5.21 ± 0.6	-	-	-	-	-
Mg – Magnesium	17.6 ± 2.0	-	-	-	-	-
Na – Sodium	99.1 ± 11.7	-	-	-	-	-
P – Phosphorus	2.06 ± 0.33	-	-	-	-	-
S – Sulphur	29.2 ± 2.1	-	-	-	-	-

Colonies were collected from the mussel farming site in the Northern Adriatic Sea, Croatia. The results are compared with literature data for other ascidian species: *Microcosmus exasperatus, Phallusia nigra*, as soft tissues [63], *Ascidia* sp. as

soft tissues [39], *Ciona intestinalis*, and *Microcosmus sulcatus* as entire tissues [64]. The results are presented as mean estimates ± standard error of the mean, where possible.
